# A *daf-7*-related TGF-β ligand (*Hc-tgh-2*) shows important regulations on the development of *Haemonchus contortus*

**DOI:** 10.1186/s13071-020-04196-x

**Published:** 2020-06-26

**Authors:** Li He, Hui Liu, Bi-Ying Zhang, Fang-Fang Li, Wen-Da Di, Chun-Qun Wang, Cai-Xian Zhou, Lu Liu, Ting-Ting Li, Ting Zhang, Rui Fang, Min Hu

**Affiliations:** State Key Laboratory of Agricultural Microbiology, Key Laboratory for the Development of Veterinary Products, Ministry of Agriculture, College of Veterinary Medicine, Huazhong Agricultural University, Wuhan, 430070 Hubei China

**Keywords:** *Haemonchus contortus*, Transforming growth factor β ligand, Development, Reproduction

## Abstract

**Background:**

In most multicellular organisms, the transforming growth factor-β (TGF-β) signalling pathway is involved in regulating the growth and stem cell differentiation. Previous studies have demonstrated the importance of three key molecules in this pathway in the parasitic nematode *Haemonchus contortus*, including one TGF-β type I receptor (*Hc-tgfbr1*), one TGF-β type II receptor (*Hc-tgfbr2*), and one co-Smad (*Hc-daf-3*), which regulated the developmental transition from the free-living to the parasitic stages of this parasite. However, almost nothing is known about the function of the TGF-β ligand (*Hc-tgh-2*) of *H. contortus*.

**Methods:**

Here, the temporal transcription profiles of *Hc-tgh-2* at eight different developmental stages and spatial expression patterns of *Hc-*TGH-2 in adult female and male worms of *H. contortus* have been examined by real-time PCR and immunohistochemistry, respectively. In addition, RNA interference (RNAi) by soaking was employed to assess the importance of *Hc-tgh-2* in the development from exsheathed third-stage larvae (xL3s) to fourth-stage larvae (L4s) in *H. contortus*.

**Results:**

*Hc-tgh-2* was continuously transcribed in all eight developmental stages of *H. contortus* studied with the highest level in the infective third-stage larvae (iL3) and *Hc*-TGH-2 was located in the muscle of the body wall, intestine, ovary of adult females and testes of adult males. Silencing *Hc-tgh-2* by the specific double-stranded RNA (dsRNA), decreased the transcript level of *Hc-tgh-2* and resulted in fewer xL3s developing to L4s *in vitro*.

**Conclusions:**

These results suggested that the TGF-β ligand, *Hc*-TGH-2, could play important roles in the developmental transition from the free-living (L3s) to the parasitic stage (L4s). Furthermore, it may also take part in the processes such as digestion, absorption, host immune response and reproductive development in *H. contortus* adults.
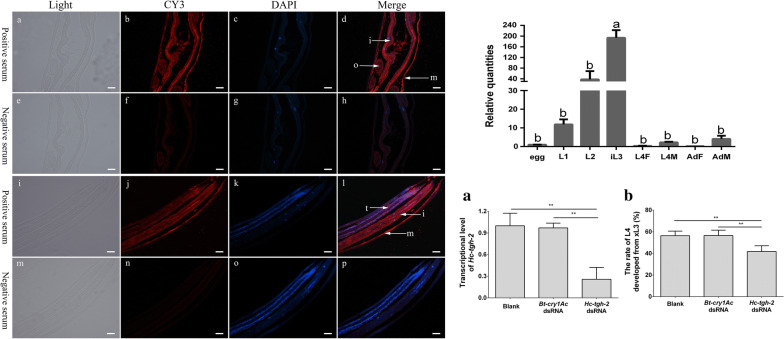

## Background

The transforming growth factor-β (TGF-β) family, consisting of more than 45 proteins with similar homo- or hetero-dimers containing a cysteine knot structural motif [1], are widely distributed in most metazoans and participate in a variety of biological processes such as development, neurological disorders and immunoregulation [2–4]. According to the structural motifs, members of the TGF-β family are divided into five groups including TGFβs, activins/inhibin, growth and differentiation factors (GDFs), bone morphogenetic proteins (BMPs) and mullerian inhibitory factor (MIF) [2, 5]. Different TGF-β members have highly specific or even opposite functions, but overlapping functions are still present among them [2, 5]. Each TGF-β protein can act as a specific ligand and bind the specific heterotetrameric receptor consisting of two TGF-β type I receptor and two TGF-β type II receptor subunits [5], therefore, different TGF-β members play diverse roles in the biological processes of multicellular animals due to specific signalling transduction.

In the free-living nematode *Caenorhabditis elegans*, there are five TGF-β ligands (*Ce*-DBL-1, *Ce*-DAF-7, *Ce*-UNC-129, *Ce*-TIG-2 and *Ce*-TIG-3) involved in regulating essential development including dauer formation, body-size determination and male tail morphology [6, 7]. However, so far, only two complete TGF-β signalling pathways (DBL-1 pathway and DAF-7 pathway) have been elucidated in detail [7, 8]. The signal transmitting from *Ce*-DBL-1 is mainly associated with body size regulation and male tail development [9, 10], in addition, it also regulates the innate immunity and lifespan [11–13]. In contrast, *Ce*-DAF-7 is expressed in ASI neurons and regulates the dauer/continuous developmental switch [14, 15]. Besides, *Ce*-DAF-7 is also involved in the regulation of longevity, reproduction and fat metabolism [16–18].

Compared with *C. elegans*, much less is known about the TGF-β ligands in parasitic nematodes. TGF-β ligand homologues were identified from a number of parasitic nematodes and limited functional studies were carried out. For example, in the filarial nematodes *Brugia malayi* and *B. pahangi*, two TGF-β homolog-1 genes (*Bm-tgh-1* and *Bp-tgh-1*, respectively) were described as members of the TGF-β family, revealing that these genes were more similar to the DBL-1 subfamily members and may play a role in the molting process in parasites inside the mammalian host [19]. Following this work, a homologue of *C. elegans daf-7* was then identified in *B. malayi* (named as *Bm-tgh-2*) [20], which was detectable over the life-cycle of this filarial parasite with the highest transcription in the microfilarial stage [20]. Subsequently, in the dog hookworm *Ancylostoma caninum*, two TGF-β ligand genes (*Ac-dbl-1* and *Ac-daf-7*) were identified and found that they were involved in regulating the male tail pattern and developmental arrest, respectively [21, 22]. Meanwhile, three homologues of *Ce*-*daf-7* were cloned from human and canine parasitic nematode *Strongyloides stercoralis* (*Ss-tgh-1*) and its two close relatives *Strongyloides ratti* (*Sr-daf-7*) and *Parastrongyloides trichosuri* (*Pt-daf-7*) and were all detected with the highest transcription in the infective third-stage larvae (iL3) [23, 24]. Regarding these three genes, *Ss-tgh-1* was then verified exclusively transcribed in the iL3 stage by RNAseq, suggesting that *Ss-tgh-1* could regulate the iL3 arrest [25]. In addition, the functional conservation of *Pt-daf-7* compared with *Ce-daf-7* was further assessed by heterologous gene rescue using a *C. elegans* mutant, revealing that *Pt-daf-7* could not rescue the *Ce-daf-7* (*e1372*) mutant strain although *Pt-daf-7* could be expressed in the ASI of *C. elegans* [26].

Recently, TGF-β ligand homologues were also identified from four trichostrongyloid nematodes (*Heligmosomoides polygyrus*, *Nippostrongylus brasiliensis*, *Teladorsagia circumcincta* and *Haemonchus contortus*). Nevertheless, functional studies have not been conducted in these nematodes except for detection of the transcript levels of these genes in their corresponding parasites [27]. More recently, in *H. contortus*, three molecules of the TGF-β signalling pathway, including one TGF-β type I receptor (*Hc-tgfbr1*), one TGF-β type II receptor (*Hc-tgfbr2*) and one co-Smad (*Hc-daf-3*), were identified to be involved in regulating the developmental transition from the free-living L3 to the parasitic stage [28–30]. In the present study, we extended the previous work and explored the functions of the TGF-β ligand homologue of *H. contortus* (*Hc-tgh-2*), aiming at illuminating its role in the regulation of the developmental processes through the TGF-β signalling pathway in *H. contortus*.

## Methods

### *Haemonchus contortus* strain and its maintenance

The *H. contortus* Haecon-5 strain was maintained in goats (3–6 months-old, helminth-free), which were infected orally with 8000–10,000 iL3s. Eggs were isolated from the faeces of infected goats, and first-stage and second-stage larvae (L1s and L2s) as well as iL3s produced by a co-culture method [31]. Fourth-stage larvae (L4s) and adults of *H. contortus* were collected from the abomasa of infected goats, euthanized with an overdose of pentobarbitone sodium (Lethobarb; Virbac Pty Ltd, Peakhurst, New South Wales, Australia) at 8 or 30 days post-infection, respectively, then all L4s and adults were extensively washed in physiological saline, and female and male worms separated prior to snap-freezing in liquid nitrogen and then stored at − 80 °C until use.

### Phylogenetic analyses of amino acid sequence data

The amino acid sequences of *Hc*-TGH-2 (GenBank: ACQ84508.1) and the homologues from 17 species (*Ancylostoma caninum*, *Ascaris suum*, *Brugia malayi*, *Caenorhabditis briggsae*, *Caenorhabditis elegans*, *Capra hircus*, *Danio rerio*, *Heligmosomoides polygyrus*, *Homo sapiens*, *Mus musculus*, *Nippostrongylus brasiliensis*, *Parastrongyloides trichosuri*, *Strongyloides stercoralis*, *Strongyloides ratti*, *Teladorsagia circumcincta, Toxocara canis* and *Trichinella spiralis*; Table 1) were aligned. Phylogenetic analyses of the aligned sequence data were conducted using the neighbor-joining (NJ), maximum parsimony (MP) and maximum likelihood (ML) methods employing the Jones–Taylor–Thornton (JTT) model [32]. Confidence limits were assessed using a bootstrap procedure employing 1000 pseudoreplicates for NJ, MP and ML in MEGA v.6.0 [32]. A 50% cut-off value was implemented for the consensus tree. A TGF-β type II receptor from *C. elegans* (GenBank: CCD63118.1) was used as the outgroup for phylogenetic analyses.

**Table 1:** Sequences used for phylogenetic analyses in the present study

Species	GenBank ID	Reference
*Ancylostoma caninum*	AAY79430.1	[21]
*Ascaris suum*	ADY41407.1	[33]
*Brugia malayi*	CDQ03041.1	[34]
*Caenorhabditis briggsae*	CAP21409.1	[35]
*Caenorhabditis elegans*	CCD61866.1	[14]
*Caenorhabditis elegans*^a^	CCD63118.1	[36]
*Capra hircus*	XP_017903600.1	[37]
*Danio rerio*	AAN03678.2	[38]
*Haemonchus contortus*	ACQ84508.1	[27]
*Heligmosomoides polygyrus*	ACR27076.1	[27]
*Homo sapiens*	NP_005802.1	[39]
*Mus musculus*	NP_034402.1	[40]
*Nippostrongylus brasiliensis*	ACR27077.1	[27]
*Parastrongyloides trichosuri*	ABQ10586.1	[23]
*Strongyloides stercoralis*	AAV84743.1	[24]
*Strongyloides ratti*	AAT79346.1	[23]
*Teladorsagia circumcincta*	ACR27078.1	[27]
*Toxocara canis*	KHN71899.1	[41]
*Trichinella spiralis*	KRY30333.1	[42]

^a^Sequence was used as the outgroup for phylogenetic analyses

### Transcriptional analyses by real-time PCR

Total RNA was isolated from individual developmental stages of *H. contortus* (eggs, L1, L2, iL3, male and female L4s, and male and female adults) using TRIzol (Life Technologies, Shanghai, China). RNA integrity and yields were verified by electrophoresis and spectrophotometric analysis (NanoDrop Technologies, Beijing, China). Complementary DNA (cDNA) was synthesized from RNA (1 μg) employing the PrimeScript™ RT reagent kit with gDNA Eraser (Perfect Real Time; Takara, Beijing, China). Nucleic acids were stored at − 80 °C until use.

According to the identified coding sequence of *Hc-tgh-2* [27] (GenBank: FJ391183), one set of primers (Hc-tgh-2-rtF/R; Additional file 1: Table S1) were designed to detect the transcriptional level of *Hc-tgh-2* in eight developmental stages of *H. contortus* by real-time PCR under the protocol as follows: 95 °C for 30 s; followed by 40 cycles at 95 °C for 15 s, 60 °C for 15 s and 72 °C for 20 s. A β-tubulin 8–9 gene (*Hc-tub8-9*) was set as a reference in all samples (in triplicate) [43] employing a set of specific primers Hc-tub8-9-rtF/R (Additional file 1: Table S1). The data of the real-time PCR were analyzed by comparing with the relative quantities of egg (egg = 1) using the 2^−∆∆Cq^ method [44]. A one-way ANOVA was used in the statistical analysis and each *P*-value was determined by *post-hoc* pairwise comparisons. This assay was repeated three times.

### Prokaryotic expression of *Hc*-TGH-2 and the localization of *Hc*-TGH-2 in *H. contortus*

According to the analysis on antigen epitopes of *Hc*-TGH-2 by the software DNAstar (http://www.dnastar.com/), one set of primers (Hc-tgh-2-eF/R) was designed to amplify the partial cDNA (151–732 bp) of *Hc-tgh-2* under the PCR cycling protocol: 95 °C for 3 min; followed by 35 cycles at 95 °C for 30 s, 60 °C for 40 s and 72 °C for 20 s; and then 72 °C for 5 min. The amplicon was inserted into the expression vector pET-28a and the construct was transformed into *E. coli* Rosseta-DE3, then the protein *Hc*-TGH-2 (51–244 aa) was expressed and purified, followed by SDS-PAGE detection. Purified recombinant *Hc*-TGH-2 protein (500 μg) was injected subcutaneously into two rabbits with multiple sites (4 immunizations with a 2-week interval between each immunization). A pre-bleed was taken from each rabbit prior to the first injection, while a final bleed was taken one week after the last immunization. Serum was treated according to a standard procedure [45]. The serum from the pre-bleed was designated as negative serum while the serum from the final bleed was designated as positive serum. All the sera were analyzed by western blot using the total protein extracted from adults of *H. contortus* with the Total Protein Extraction Kit (Bestbio Company, Guangzhou, China).

The serum was used to detect the expression patterns of *Hc-*TGH-2 in adult males and females of *H. contortus* by immunohistochemistry, respectively, as previously described [29]. In brief, approximately 50 *H. contortus* adult males or females were fixed in 4% paraformaldehyde (Biosharp, Hefei, China) at 4 °C, respectively. Then the single worm was dehydrated in a graded ethanol series (75% for 4 h, 85% for 2 h, 90% for 2 h, 95% for 1 h once and 100% twice for 30 min) sequentially, followed by embedding in paraffin. Sections (4 μm) were cut and flattened on polylysine slides, followed by paraffinating (xylene treated twice for 20 min) and rehydrating in a series of graded ethanol (100% twice for 10 min; 95% once for 5 min, 90% once for 5 min, 80% once for 5 min, 70% once for 5 min each), then washed with phosphate buffer solution (PBS) for three times (5 min). Antigens were recovered by the microwave, then endogenous catalase was eliminated by 3% hydrogen peroxide. The sections were washed with PBS three times (5 min), then blocked with 5% bovine serum albumin (BSA) for 20 min in a humidified chamber. The sections were incubated with approximately 50 μl polyclonal anti-*Hc*-TGH-2 antibody (positive serum) or negative serum (each at 1:100 dilution) at 4 °C overnight, respectively. The serum was removed and the sections were washed three times with PBS (5 min). Then the sections were incubated at 37 °C for 50 min in anti-rabbit immunoglobulin (IgG) (raised in sheep) conjugated with fluorescein (Aspen, Chengdu, China) in the dark. The sections were then washed with PBS for three times (5 min) to remove the secondary antibody. After that, the sections were incubated at room temperature for 5 min in 4, 6-diamidino-2-phenylindole (DAPI) solution in the dark. The sections were washed in PBS three times (5 min) again and then assessed in detail using an epifluorescence microscope (Olympus CX-21; Olympus, Shenzhen, China). All images were processed using Adobe Photoshop CS 6.0.

### Preparation of double-stranded RNA and RNA interference (RNAi) in *H. contortus*

Two sets of specific PCR primers (Hc-tgh-2-sF1/sR1 and Hc-tgh-2-sF2/sR2) were designed to amplify the coding sequences of the *Hc*-TGH-2 functional domain (864 bp) for constructing two plasmids that were used to synthesize specific dsRNA (Additional file 1: Table S1) as previously described [28, 29]. One set of primers (Hc-tgh-2-sF1/sR1) was tagged with the T7 promoter site in forward direction and restriction enzyme *Bam*H I site in reverse direction, respectively. Another set of primers (Hc-tgh-2-sF2/sR2) was tagged with the restriction enzyme *Bam*H I site in forward direction and the T7 promoter site in reverse direction, respectively. The sequences of the *Hc-*TGH-2 functional domain (864 bp) were amplified by these two sets of specific PCR primers under the same cycling conditions: 95 °C for 5 min; followed by 35 cycles of 95 °C for 30 s, 55.4 °C for 30 s, 72 °C for 2 min; and then 72 °C for 5 min. PCR products were then inserted into pTOPO-Blunt Simple vector (Aidlab Biotechnologies Co., Ltd, Beijing, China) by a ClonExpress^TM^ II One Step Cloning Kit (Vazyme Biotech Co., Ltd, Nanjing, China), respectively. The irrelevant control was a *cry1Ac* gene from *Bacillus thuringiensis* (*Bt-cry1Ac*, GenBank: GU322939.1), which has no homology with any *H. contortus* gene [28, 29]. The sequence of *Bt-cry1Ac* was cloned using two sets of specific PCR primers Bt-cry1Ac-sF1/sR1 and Bt-cry1Ac-sF2/sR2 (Additional file 1: Table S1) under the following cycling protocol: 95 °C for 5 min; followed by 35 cycles of 95 °C for 30 s, 55 °C for 30 s, 72 °C for 1 min; then 72 °C for 5 min. PCR products were then inserted into pTOPO-Blunt Simple vector, as previously described. All constructs were extracted using plasmid Maxi Kit (Aidlab Biotechnologies Co., Ltd) and then were estimated spectrophotometrically (NanoDrop Technologies) and stored at − 20 °C until use, respectively. The restriction enzyme *Bam*HI was used to linearize the constructs containing *Hc-tgh-2* or *Bt-cry1Ac* fragments, respectively, and the linearized templates were verified by electrophoresis and spectrophotometric analysis (NanoDrop Technologies) and used to synthesize single-stranded RNA (ssRNA) using a RNA large-scale T7 production system according to the instruction manual of MEGAscript® T7 Transcription Kit (Ambion, Shanghai, China). An equal quantity (~ 500 µg) of sense ssRNA and antisense ssRNA were used to synthesize dsRNA with the treatment of 5× annealing buffer at room temperature for 2 h. The quality and yield of ssRNA and dsRNA were verified by electrophoresis and spectrophotometric analysis (NanoDrop Technologies), respectively. All RNA was immediately frozen and stored at − 80 °C until use.

The RNA interference assay was performed as previously described [28]. Briefly, *H. contortus* L3s collected from faecal culture were exsheathed in 0.1% sodium hypochlorite/PBS for 30 min at 38 °C, followed by washing twice in sterile PBS by centrifugation at 600×*g* (5 min) at 23 °C and four times in PBS containing antibiotic-antimycotic solution (Gibco, Shanghai, China). Then, the exsheathed L3s (xL3s) were suspended in Earle’s Balanced Salt Solution (EBSS, pH adjusted to 5.2; Sigma-Aldrich, Shanghai, China) with antibiotic-antimycotic solution (Gibco) with a final concentration at 33,000 xL3/ml and incubated with different dsRNA (1 mg/ml). Before incubation, the *Hc-tgh-2* dsRNA, *Bt-cry1Ac*-specific dsRNA (irrelevant control) or nuclease-free water (blank control) were pre-incubated (separately) with RNasin (8 U) and Lipofectin Reagent (Invitrogen, Shanghai, China) for 10 min at 25 °C (room temperature), respectively, and added into 30 μl of EBSS cultures containing xL3s. After incubation at 37 °C in 20% CO_2_ for 24 h, 300 larvae were transferred to 100 μl of fresh EBSS without dsRNA (in triplicate) and cultured for another 7 days and the replicate cultures were examined by microscopy to count the numbers of xL3s and L4s according to the morphological changes of the buccal capsule [28, 46, 47]. The remaining larvae were collected to isolate RNA for detecting the transcriptional change of the gene *Hc-tgh-2* by real-time PCR with one set of primers (Hc-tgh-2-rtF/R; Additional file 1: Table S1). *18S* rRNA was used as a reference marker [48] and the sequences of primers Hc-18s-rtF/R are shown in Additional file 1: Table S1. The PCR cycling protocol used was: 95 °C for 30 s, followed by 40 cycles at 95 °C for 15 s, 60 °C for 15 s and 72 °C for 20 s. The efficiency of the PCR was calculated using an established formula and the real-time PCR data was subjected to analysis using the 2^−∆∆Cq^ method [44].

## Results

### Phylogenetic analyses of amino acid sequence data

**Fig. 1:**
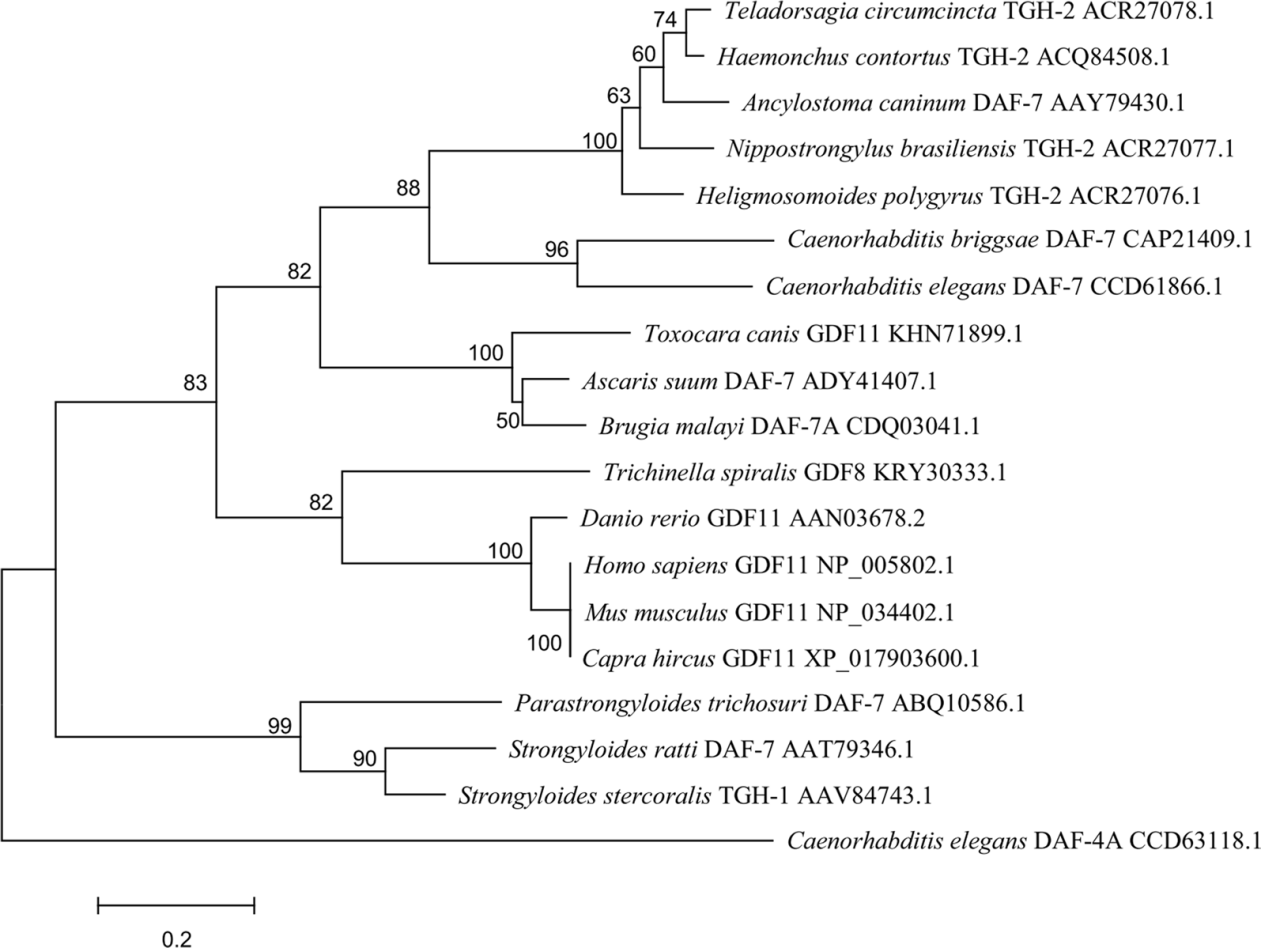
Phylogenetic relationship of *Haemonchus contortus Hc*-TGH-2 (ACQ84508.1) with other TGF-β ligand homologues. The homologues were from 17 species including *Ancylostoma caninum* (AAY79430.1), *Ascaris suum* (ADY41407.1), *Brugia malayi* (CDQ03041.1), *Caenorhabditis briggsae* (CAP21409.1), *Caenorhabditis elegans* (CCD61866.1), *Capra hircus* (XP_017903600.1), *Danio rerio* (AAN03678.2), *Heligmosomoides polygyrus* (ACR27076.1), *Homo sapiens* (NP_005802.1), *Mus musculus* (NP_034402.1), *Nippostrongylus brasiliensis* (ACR27077.1), *Parastrongyloides trichosuri* (ABQ10586.1), *Strongyloides stercoralis* (AAV84743.1), *Strongyloides ratti* (AAT79346.1), *Teladorsagia circumcincta* (ACR27078.1), *Toxocara canis* (KHN71899.1) and *Trichinella spiralis* (KRY30333.1), a TGF-β type II receptor from *C. elegans* (CCD63118.1) was used as the outgroup for this phylogenetic analysis. Bootstrap values are shown above or below the branches

The amino acid sequence of the TGF-β ligand homologue of *H. contortus* (designated as *Hc*-TGH-2) was obtained from GenBank (accession number ACQ84508.1 [27]). Phylogenetic analyses showed that the topologies of the MP, ML and NJ trees were concordant. *Hc*-TGH-2 grouped together with homologues from four strongylid parasitic nematodes including *Ancylostoma caninum*, *Heligmosomoides polygyrus*, *Nippostrongylus brasiliensis* and *Teladorsagia circumcincta* with 100% nodal support, which formed a cluster (88%) with DAF-7 homologues of two *Caenorhabditis* spp. (Fig. 1). This big cluster and another smaller cluster (100%) containing three TGF-β ligand homologues from two parasites of the Ascaridata (*Ascaris suum* and *Toxocara canis*) and one parasite of the Filarioidea (*Brugia malayi*) grouped together with 82% nodal support (Fig. 1). One TGF-β ligand homologue from *Trichinella spiralis* formed a cluster (82%) with four TGF-β ligand homologues from four metazoans (*Capra hircus*, *Danio rerio*, *Homo sapiens* and *Mus musculus*), which grouped together with 10 selected TGF-β ligand homologues from strongylid, ascarid and filarial nematodes by 83% nodal support, to the exclusion of TGF-β ligand homologues of three parasitic nematodes (*Parastrongyloides trichosuri*, *Strongyloides stercoralis* and *Strongyloides ratti*) that have an alternative free-living life-cycle (Fig. 1).

### Transcriptional analysis of *Hc-tgh-2* in eight different developmental stages of *H. contortus*

**Fig. 2:**
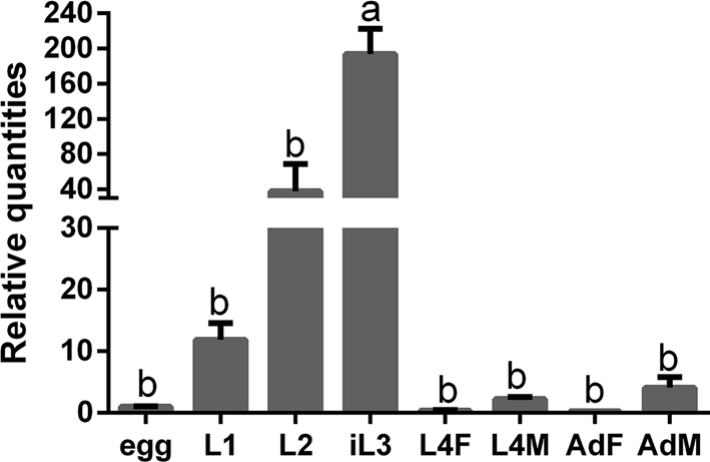
Transcriptional levels of the *Hc-tgh-2* gene in eight developmental stages of *Haemonchus contortus*. The relative quantities (compared with egg, egg = 1) are shown as mean values (± standard error of the mean, SE). *Abbreviations*: Egg, eggs; L1, first-stage larvae; L2, second-stage larvae; iL3, infective third-stage larvae; L4F, females of fourth-stage larvae; L4M, males of fourth-stage larvae; AdF, adult females; AdM, adult males. The statistical analysis showed that ANOVA, *F*_(7, 16)_ = 20.11002, *P* < 0.0001. Egg *vs* L1, *P* = 0.9994; Egg *vs* L2, *P* = 0.6577; Egg *vs* iL3, *P* < 0.0001; Egg *vs* L4F, *P* > 0.9999; Egg *vs* L4M, *P* > 0.9999; Egg *vs* AdF, *P* > 0.9999; Egg *vs* AdM, *P* > 0.9999; L1 *vs* L2, *P* = 0.9090; L1 *vs* iL3, *P* < 0.0001; L1 *vs* L4F, *P* = 0.9991; L1 *vs* L4F, *P* = 0.9997; L1 *vs* AdF, *P* = 0.9990; L1 *vs* AdM, *P* > 0.9999; L2 *vs* iL3, *P* < 0.0001; L2 *vs* L4F, *P* = 0.6399; L2 *vs* L4F, *P* = 0.6924; L2 *vs* AdF, *P* = 0.6358; L2 *vs* AdM, *P* = 0.7419; iL3 *vs* L4F, *P* < 0.0001; iL3 *vs* L4M, *P* < 0.0001; iL3 *vs* AdF, *P* < 0.0001; iL3 *vs* AdM, *P* < 0.0001; L4F *vs* L4M, *P* > 0.9999; L4F *vs* AdF, *P* > 0.9999; L4F *vs* AdM, *P* > 0.9999; L4M *vs* AdF, *P* > 0.9999; L4M *vs* AdM, *P* > 0.9999; AdF *vs* AdM, *P* > 0.9999. There were significant differences between stages indicated by different capital letters (a, b) (*P* < 0.01)

The relative transcripts of *Hc-tgh-2* were detectable in eight different developmental stages (eggs, L1s, L2s, iL3s, female L4s, male L4s, adult females and adult males) of *H. contortus* (Fig. 2). The transcript abundance of *Hc-tgh-2* was highest at iL3 stage, with significant differences compared with other developmental stages (*F*_(7, 16)_ = 20.11002, *P* < 0.0001, respectively) (Fig. 2). There was no statistically significant difference among all other developmental stages except for iL3 (Fig. 2).

### Localization of *Hc-*TGH-2 in *H. contortus* adults

**Fig. 3:**
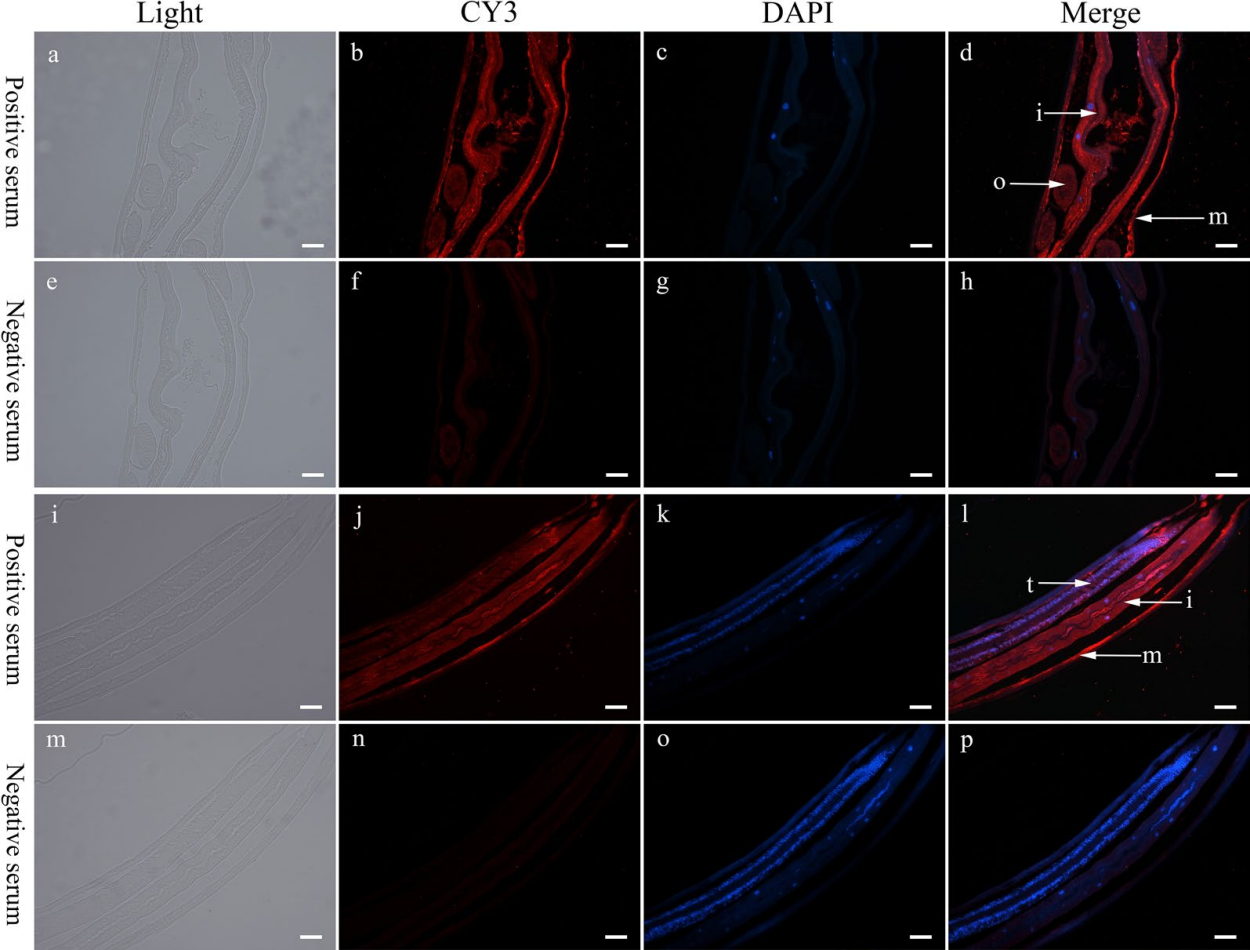
The expression patterns of *Hc*-TGH-2 in *Haemonchus contortus* adults. **a**–**h**, the expression pattern of *Hc*-TGH-2 in *H. contortus* adult females with an exposure time of 2500 ms. **i**–**p** the expression pattern of *Hc*-TGH-2 in *H. contortus* adult males, with an exposure time of 3000 ms. Positive serum was the serum from the final bleed after the last immunization (containing the antibody against recombinant *Hc*-TGH-2), negative serum is the serum from the pre-bleed before the first immunization (without the antibody of *Hc*-TGH-2). *Abbreviations*: i, intestine; o, ovaries; m, muscles of the body wall; t: testes. *Scale-bars*: 50 μm

The coding sequence of truncated *Hc*-TGH-2 (51–244 aa, 29 KD) was 582 bp in length and expressed in *E. coli* Rosseta-DE3 (Additional file 2: Figure S1). The polyclonal antibody against r*Hc*-TGH-2 produced by immunizing rabbits could bind to the native *Hc*-TGH-2 specifically (Additional file 3: Figure S2). Using this polyclonal antibody as a probe in the immunobiochemical assay, *Hc*-TGH-2 was detected in the muscle of the body wall, intestine and ovaries of *H. contortus* adult females (Fig. 3a–h). In adult males, *Hc*-TGH-2 was expressed in the muscle of the body wall, intestine and testes (Fig. 3i–p).

### Effect of silencing *Hc-tgh-2* on the development of *H. contortus* xL3 *in vitro*

**Fig. 4:**
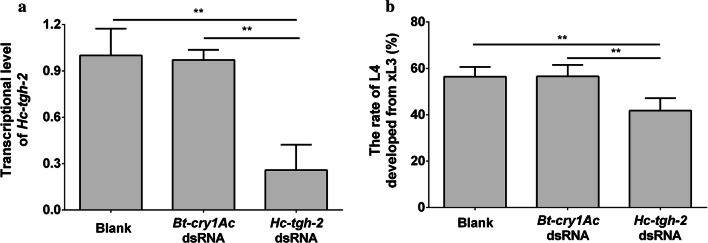
Effect of *Hc*-*tgh-2* dsRNA treatment on the xL3 development of *Haemonchus contortus in vitro*. **a** The transcriptional changes of *Hc-tgh-2* in *H. contortus* after RNAi detected by real-time PCR. **b** The ratios of L4s developed from xL3s *in vitro* for another 7 days after RNAi. **P < 0.01

After xL3 was soaked in *Hc-tgh-2* dsRNA for 24 h, the transcriptional level of *Hc-tgh-2* was significantly decreased compared with the blank control (no dsRNA) and irrelevant dsRNA control group (*Bt-cry1Ac* dsRNA) (*F*_(2, 6)_ = 25.95825, *P* = 0.0021 and *P* = 0.0026, respectively), and there was no statistically significant difference between the two control groups (Fig. 4a). In addition, silencing *Hc-tgh-2* resulted in fewer xL3s developing to L4s *in vitro* after incubation for another 7 days compared with any control groups (*F*_(2, 10)_ = 13.01698, *P* = 0.0035 and *P* = 0.0033, respectively) (Fig. 4b).

## Discussion

In the present study, extending from our previous work on molecules in the TGF-β pathway [28–30], the genetic relationship of a predicted TGF-β ligand of *H. contortus* (*Hc*-TGH-2) with selected homologues from different metazoan species were analysed. In addition, the temporal transcriptional and spatial expression profiles of its coding gene (*Hc*-*tgh-2*) were explored and its importance in controlling the development from xL3s (free-living stage) to L4s (parasitic stage) was also investigated.

Phylogenetic analyses revealed that *Hc*-TGH-2 was closer to the DAF-7 homologues of two *Caenorhabditis spp.* than the TGF-β ligands from metazoans, which is different from the phylogenetic relationships of TGF-β receptors from *H. contortus* (*Hc*-TGFBR1 and *Hc*-TGFBR2) being more distant from those of *Caenorhabditis* spp. and metazoans [28, 29]. It is likely that the molecules of the TGF-β signalling pathway from *H. contortus* were divergent along with the evolution of this pathway in parasitic nematodes. It is proposed that the TGF-β signalling pathway in parasitic nematodes played a role in maintaining the arrested state [49, 50], however, the function of TGF-β ligand in different parasitic nematodes may be different due to the divergent conservation of the amino acid sequences, especially for those from *P. trichosuri*, *S. stercoralis* and *S. ratti* as they did not group with the TGF-β ligands of other selected nematodes.

In the present study, the transcripts of *Hc-tgh-2* were detected at eight different developmental stages, the results showed that the transcriptional profiles of *Hc-tgh-2* in eggs, iL3s, L4s and adults were consistent with a previous report [27] and the transcriptional profiles of *Hc-tgh-2* at other developmental stages (L1s and L2s) were supplemented. In *H. contortus*, the transcripts of *Hc-tgh-2* were detected in eggs at a lower level and reached a peak in iL3s (arrested stage), which is similar to the transcriptional profile of TGF-β ligands from some parasitic nematodes including *A. caninum* [21, 22], *N. brasiliensis* [27], *P. trichosuri* [23], *S. stercoralis* [24] and *S. ratti* [23], but different from that of *Ce-daf-7* in *C. elegans* [14]. In *C. elegans*, the transcript abundance of *Ce-daf-7* is highest in the L1 stage (prior to the arrested stage) and lowest in the dauer stage, and it is deemed that the TGF-β signalling pathway controls the dauer/reproductive developmental choice in *C. elegans* [14]. Nevertheless, the arrested stage (iL3s) is inevitable in parasitic nematodes, thus it may not be necessary for parasitic nematodes to regulate the process of entry into the arrested stage. The highest transcript levels of TGF-β ligands in iL3s of parasitic nematodes suggested that the TGF-β signalling pathway perhaps plays an important role in the steps behind the process of entering into the arrested stage, which means that the TGF-β signalling pathway may prefer to maintain the arrested stage and/or regulate the recovery of development. It has been presumed that the recovery of the development of iL3s is depended on the insulin-like signalling pathway, but not the TGF-β signalling pathway [51], thus parasitologists prefer to believe that the role of the TGF-β signalling pathway is maintaining the arrested stage [49, 50]. However, this viewpoint may not be broadly applied as the transcript levels of TGF-β ligands in *T. circumcincta* and *H. polygyrus* are not highest in iL3s, but peak in adults and eggs respectively [27]. Furthermore, even in *H. contortus*, the transcription levels of the TGF-β type I receptor gene *Hc-tgfbr1* was highest in adult females [28]. Taken together, we propose that the functions of the TGF-β signalling pathway may have arisen some changes or divergence in the evolution of parasitic nematodes. The mechanisms need to be investigated in the future.

In our opinion, the TGF-β signalling pathway may prefer to regulate the recovery of development in the arrested stage (iL3s) in *H. contortus* on account of the decreased proportion of *H. contortus* L4s developed from xL3 with downregulated *Hc-tgh-2* induced by RNAi *in vitro*, which is similar to the results of other three key molecules of the TGF-β signalling pathway in *H. contortus* studied recently, including a TGF-β type I receptor (*Hc-tgfbr1*) [28], a TGF-β type II receptor (*Hc-tgfbr2*) [29] and a co-Smad (*Hc-daf-3*) [30]. This is different from the functions of this signaling in *A. caninum* [51]. Both *H. contortus* and *A. caninum* are Strongylida nematodes of clade V [52], however, these two parasitic nematodes have different routes to infect the appropriate hosts. A majority (85%) of *A. caninum* L3s infect the hosts *via* the percutaneous route and resume feeding within 48 h post-infection, a minority (15%) of *A. caninum* L3s can infect hosts orally and most larvae could only resume feeding until they develop to L4s [53]. The resumption of feeding was used as a marker for studying the developmental resumption in *A. caninum* after the parasites enter into the host [51]. However, in *H. contortus*, L3s can only infect the host *via* the oral route and are unable to resume feeding until the L3s develop to L4s [54], which means that feeding resumption may not be a good marker for studying the developmental resumption of L3s in *H. contortus*. Therefore, we propose that the TGF-β signalling pathway in different parasitic nematodes (for example, *A. caninum* or *H. contortus*) may function differently in the developmental recovery. This needs to be investigated in the future by *in vivo* analysis of RNAi treated parasites as previously described [55], which will provide some ideas for controlling *H. contortus*.

In *H. contortus*, the muscles of the body wall act as storage for glycogen, phospholipids and neutral lipids [56], thus the *Hc-tgh-2* may be involved in storage for glycogen, phospholipids and neutral lipids due to its expression in the muscle cells of the body wall in adult worms. The body wall is the first line of *H. contortus* to be contacted by the host environment, thus, it is likely that *Hc-tgh-2* may be related to the survival of *H. contortus* in the host. A homologue of TGF-β from *B. malayi* (Bm-TGH-2) shows a low level of binding ability with mammalian receptors [20]. A TGF-β Mimic (TGM) of the murine parasitic nematode *Heligmosomoides polygyrus* was identified to act as a cytokine to exploit an endogenous pathway of immunoregulation in the host [57] and two other TGM members of *H. polygyrus* show an active function in an TGF-β bioassay (cell line clone MFB-F11) that can induct T cell Foxp3 expression [58]. Therefore, the TGF-β family of parasitic nematodes may take part in immunoregulation in their host.

In *H. contortus* adult females and males, *Hc*-TGH-2 was also strongly expressed in the intestine, which is an important organ for the digestive system and a good source for mucosal candidate antigens to induce an immune response in the host [59, 60], suggesting that *Hc-tgh-2* perhaps plays a role in digestion, absorption and host immune response. Furthermore, *Hc-tgh-2* may also be involved in the reproductive development due to the strong expression in gonads (ovaries in adult females and testes in adult males) of *H. contortus*. In *C. elegans*, the *daf-7* mutant worms showed an egg-laying defect and *Ce-daf-7* is identified as a regulator that can expanse the larval germline progenitor pool [7, 18]. Furthermore, phylogenetic analyses showed that *Hc*-TGH-2 was close to *Ce*-DAF-7 of *C. elegans*, so we propose that *Hc-tgh-2* may regulate the germline proliferation and differentiation and the behavior of egg-laying in *H. contortus*. Another key molecule of the TGF-β signalling pathway, *Hc*-TGFBR2, also strongly expressed in the intestine and gonads of *H. contortus* adults [29], implying that the TGF-β signalling pathway may play important roles in the digestive and reproductive systems in adults to facilitate the parasites survival and reproduction in the host.

## Conclusions

In the present study, a *daf-7*-related TGF-β ligand, *Hc-tgh-2*, was characterized from the parasitic nematode *H. contortus*. *Hc-tgh-2* was continuously transcribed in eight developmental stages of *H. contortus* with the highest level in iL3s, and the decreased transcription of *Hc-tgh-2* in xL3s induced by the specific dsRNA could retard the development of xL3 to L4s *in vitro*, suggesting that *Hc-tgh-2* could regulate the development from xL3 to L4 in *H. contortus*. The immunohistochemical results indicated that *Hc*-TGH-2 was expressed in the muscle of the body wall, intestine and gonads of adult stages of *H. contortus*, suggesting that *Hc-tgh-2* may play important roles in the digestive and reproductive systems in *H. contortus* adults. Taken together, TGF-β *Hc*-TGH-2 could play an important role in the transition from the free-living (L3s) to the parasitic stage (L4), digestion, absorption, host immune response and reproductive development in *H. contortus*.

## Supplementary information

**Additional file 1: Table S1.** Oligonucleotide primers (5’-3’) used in the present study.

**Additional file 2: Figure S1.** Prokaryotic expression and purification of *Hc*-TGH-2, as analyzed by SDS-PAGE. Lane 1: protein marker; Lane 2: purified recombinant protein *Hc*-TGH-2; Lane 3: recombinant protein expression non-induced by IPTG; Lane 4: recombinant protein *Hc*-TGH-2 expression induced by IPTG.

**Additional file 3: Figure S2.** Western blot analysis detecting the expression of native *Hc*-TGH-2 in *Haemonchus contortus* adult worms. The polyclonal antibody of recombinant *Hc*-TGH-2 protein were analyzed by western blot using the total protein of *Haemonchus contortus* adults. Lane 1: negative serum without the antibody against recombinant *Hc*-TGH-2; Lane 2: positive serum with the antibody against recombinant *Hc*-TGH-2.

## Data Availability

Data supporting the conclusions of this article are included within the article and its additional files.

## Abbreviations

BSA: bovine serum albumin
DAPI: 4, 6-diamidino-2-phenylindole
dsRNA: double-stranded RNA
EBSS: Earle’s Balanced Salt Solution
ML: maximum likelihood
MP: maximum parsimony
NJ: neighbour-joining
PBS: phosphate buffer solution
SDS-PAGE: sodium dodecyl sulfate polyacrylamide gel electrophoresis
ssRNA: single-stranded RNA

## References

[1] Galat A (2011). Common structural traits for cystine knot domain of the TGF beta superfamily of proteins and three-fingered ectodomain of their cellular receptors. Cell Mol Life Sci..

[2] Herpin A, Lelong C, Favrel P (2004). Transforming growth factor-beta-related proteins: an ancestral and widespread superfamily of cytokines in metazoans. Dev Comp Immunol..

[3] Chen W, Ten Dijke P (2016). Immunoregulation by members of the TGF beta superfamily. Nat Rev Immunol..

[4] Kashima R, Hata A (2018). The role of TGF-beta superfamily signaling in neurological disorders. Acta Biochim Biophys Sinica..

[5] Santibañez JF, Quintanilla M, Bernabeu C (2011). TGF-beta/TGF-beta receptor system and its role in physiological and pathological conditions. Clin Sci (Lond)..

[6] Patterson GI, Padgett RW (2000). TGFβ-related pathways. Roles in *Caenorhabditis elegans* development. Trends Genet..

[7] Gumienny TL, Savage-Dunn C (2013). TGF-β signaling in *C. elegans*. Wormbook.

[8] Savage-Dunn C, Padgett RW (2017). The TGF-β family in *Caenorhabditis elegans*. Cold Spring Harb Perspect Biol..

[9] Morita K, Chow KL, Ueno N (1999). Regulation of body length and male tail ray pattern formation of *Caenorhabditis elegans* by a member of TGF-beta family. Development..

[10] Suzuki Y, Yandell MD, Roy PJ, Krishna S, Savage-Dunn C, Ross RM (1999). A BMP homolog acts as a dose-dependent regulator of body size and male tail patterning in *Caenorhabditis elegans*. Development..

[11] Zhang X, Zhang Y (2009). Neural-immune communication in *Caenorhabditis elegans*. Cell Host Microbe..

[12] Roberts AF, Gumienny TL, Gleason RJ, Wang H, Padgett RW (2010). Regulation of genes affecting body size and innate immunity by the DBL-1/BMP-like pathway in *Caenorhabditis elegans*. BMC Dev Biol..

[13] So S, Tokumaru T, Miyahara K, Ohshima Y (2011). Control of lifespan by food bacteria, nutrient limitation and pathogenicity of food in *C. elegans*. Mech Ageing Dev..

[14] Ren P, Lim CS, Johnsen R, Albert PS, Pilgrim D, Riddle DL (1996). Control of *C. elegans* larval development by neuronal expression of a TGF-beta homolog. Science..

[15] Schackwitz WS, Inoue T, Thomas JH (1996). Chemosensory neurons function in parallel to mediate a pheromone response in *C. elegans*. Neuron..

[16] Shaw WM, Luo S, Landis J, Ashraf J, Murphy CT (2007). The *C. elegans* TGF-beta dauer pathway regulates longevity *via* insulin signaling. Curr Biol..

[17] Greer ER, Pérez CL, Gilst MRV, Lee BH, Ashrafi K (2008). Neural and molecular dissection of a *C. elegans* sensory circuit that regulates fat and feeding. Cell Metab..

[18] Dalfó D, Michaelson D, Hubbard EJ (2012). Sensory regulation of the *C. elegans* germline through TGF-beta-dependent signaling in the niche. Curr Biol..

[19] Gomez-Escobar N, Lewis E, Maizels RM (1998). A novel member of the transforming growth factor-beta (TGF-beta) superfamily from the filarial nematodes *Brugia malayi* and *B. pahangi*. Exp Parasitol..

[20] Gomez-Escobar N, Gregory WF, Maizels RM (2000). Identification of *tgh-2*, a filarial nematode homolog of *Caenorhabditis elegans daf-7* and human transforming growth factor beta, expressed in microfilarial and adult stages of *Brugia malayi*. Infect Immun..

[21] Brand AM, Varghese G, Majewski W, Hawdon JM (2005). Identification of a DAF-7 ortholog from the hookworm *Ancylostoma caninum*. Int J Parasitol..

[22] Freitas TC, Arasu P (2005). Cloning and characterisation of genes encoding two transforming growth factor-beta-like ligands from the hookworm, *Ancylostoma caninum*. Int J Parasitol..

[23] Crook M, Thompson FJ, Grant WN, Viney ME (2005). *daf-7* and the development of *Strongyloides ratti* and *Parastrongyloides trichosuri*. Mol Biochem Parasitol..

[24] Massey HC, Castelletto ML, Bhopale VM, Schad GA, Lok JB (2005). *Sst-tgh-1* from *Strongyloides stercoralis* encodes a proposed ortholog of *daf-7* in *Caenorhabditis elegans*. Mol Biochem Parasitol..

[25] Stoltzfus JD, Minot S, Berriman M, Nolan TJ, Lok JB (2012). RNAseq analysis of the parasitic nematode *Strongyloides stercoralis* reveals divergent regulation of canonical dauer pathways. PLoS Negl Trop Dis..

[26] Crook M, Grant K, Grant WN (2010). Failure of *Parastrongyloides trichosuri daf-7* to complement a *Caenorhabditis elegans daf-7* (e1372) mutant: implications for the evolution of parasitism. Int J Parasitol..

[27] McSorley HJ, Grainger JR, Harcus Y, Murray J, Nisbet AJ, Knox DP (2010). *daf-7*-related TGF-beta homologues from trichostrongyloid nematodes show contrasting life-cycle expression patterns. Parasitology..

[28] He L, Gasser RB, Korhonen PK, Di W, Li F, Zhang H (2018). A TGF-beta type I receptor-like molecule with a key functional role in *Haemonchus contortus* development. Int J Parasitol..

[29] He L, Gasser RB, Li T, Di W, Li F, Zhang H (2019). A TGF-beta type II receptor that associates with developmental transition in *Haemonchus contortus* in vitro. PLoS Negl Trop Dis..

[30] Di W, Liu L, Zhang T, Li F, He L, Wang C (2019). A DAF-3 co-Smad molecule functions in *Haemonchus contortus* development. Parasit Vectors..

[31] Zawadzki JL, Kotze AC, Fritz JA, Johnson NM, Hemsworth JE, Hines BM (2012). Silencing of essential genes by RNA interference in *Haemonchus contortus*. Parasitology..

[32] Tamura K, Stecher G, Peterson D, Filipski A, Kumar S (2013). MEGA6: molecular evolutionary genetics analysis version 6.0. Mol Biol Evol..

[33] Wang J, Czech B, Crunk A, Wallace A, Mitreva M, Hannon GJ (2011). Deep small RNA sequencing from the nematode *Ascaris* reveals conservation, functional diversification, and novel developmental profiles. Genome Res..

[34] Ghedin E, Wang S, Spiro D, Caler E, Zhao Q, Crabtree J (2007). Draft genome of the filarial nematode parasite *Brugia malayi*. Science..

[35] Stein LD, Bao Z, Blasiar D, Blumenthal T, Brent MR, Chen N (2003). The genome sequence of *Caenorhabditis briggsae*: a platform for comparative genomics. PLoS Biol..

[36] Gunther CV, Georgi LL, Riddle DL (2000). A *Caenorhabditis elegans* type I TGFβ receptor can function in the absence of type II kinase to promote larval development. Development..

[37] Lowe TM, Eddy SR (1997). tRNAscan-SE: a program for improved detection of transfer RNA genes in genomic sequence. Nucleic Acids Res..

[38] Biga PR, Roberts SB, Iliev DB, McCauley LA, Moon JS, Collodi P (2005). The isolation, characterization, and expression of a novel GDF11 gene and a second myostatin form in zebrafish, *Danio rerio*. Comp Biochem Physiol B Biochem Mol Biol..

[39] Gamer LW, Wolfman NM, Celeste AJ, Hattersley G, Hewick R, Rosen V (1999). A novel BMP expressed in developing mouse limb, spinal cord, and tail bud is a potent mesoderm inducer in *Xenopus embryos*. Dev Biol..

[40] Nakashima M, Toyono T, Akamine A, Joyner A (1999). Expression of growth/differentiation factor 11, a new member of the BMP/TGFbeta superfamily during mouse embryogenesis. Mech Dev..

[41] Zhu XQ, Korhonen PK, Cai H, Young ND, Nejsum P, von Samson-Himmelstjerna G (2015). Genetic blueprint of the zoonotic pathogen *Toxocara canis*. Nat Commun..

[42] Korhonen PK, Pozio E, La Rosa G, Chang BC, Koehler AV, Hoberg EP (2016). Phylogenomic and biogeographic reconstruction of the *Trichinella* complex. Nat Commun..

[43] Guo X, Zhang H, Zheng X, Zhou Q, Yang Y, Chen X (2016). Structural and functional characterization of a novel gene, *Hc-daf-22*, from the strongylid nematode *Haemonchus contortus*. Parasit Vectors..

[44] Pfaffl MW (2001). A new mathematical model for relative quantification in real-time RT-PCR. Nucleic Acids Res..

[45] Wright K (1989). Antibodies a Laboratory Manual by E Harlow and D Lane. Biochem Mol Biol Educ..

[46] Sommerville RI (1966). The development of *Haemonchus contortus* to the fourth stage in vitro. J Parasitol..

[47] Mapes CJ (1969). The development of *Haemonchus contortus in vitro*. Parasitology..

[48] Kotze AC, Bagnall NH (2006). RNA interference in *Haemonchus contortus*: suppression of beta-tubulin gene expression in L3, L4 and adult worms in vitro. Mol Biochem Parasitol..

[49] Crook M (2014). The dauer hypothesis and the evolution of parasitism: 20 years on and still going strong. Int J Parasitol..

[50] Viney ME, Thompson FJ, Crook M (2005). TGF-beta and the evolution of nematode parasitism. Int J Parasitol..

[51] Tissenbaum HA, Hawdon J, Perregaux M, Hotez P, Guarente L, Ruvkun G (2000). A common muscarinic pathway for diapause recovery in the distantly related nematode species *Caenorhabditis elegans* and *Ancylostoma caninum*. Proc Natl Acad Sci USA.

[52] Blaxter ML, De Ley P, Garey JR, Liu LX, Scheldeman P, Vierstraete A (1998). A molecular evolutionary framework for the phylum Nematoda. Nature..

[53] Hawdon JM, Volk SW, Rose R, Pritchard DI, Behnke JM, Schad GA (1993). Observations on the feeding behaviour of parasitic third-stage hookworm larvae. Parasitology..

[54] Gamble HR, Mansfield LS (1996). Characterization of excretory-secretory products from larval stages of *Haemonchus contortus* cultured in vitro. Vet Parasitol..

[55] Samarasinghe B, Knox DP, Britton C (2011). Factors affecting susceptibility to RNA interference in *Haemonchus contortus* and in vivo silencing of an H11 aminopeptidase gene. Int J Parasitol..

[56] Sood ML, Kalra S (1977). Histochemical studies on the body wall of nematodes: *Haemonchus contortus* (Rud., 1803) and *Xiphinema insigne* Loos, 1949. Z Parasitenkd..

[57] Johnston CJC, Smyth DJ, Kodali RB, White MPJ, Harcus Y, Filbey KJ (2017). A structurally distinct TGF-beta mimic from an intestinal helminth parasite potently induces regulatory T cells. Nat commun..

[58] Smyth DJ, Harcus Y, White MPJ, Gregory WF, Nahler J, Stephens I (2018). TGF-beta mimic proteins form an extended gene family in the murine parasite *Heligmosomoides polygyrus*. Int J Parasitol..

[59] Jasmer DP, Lahmers KK, Brown WC (2007). *Haemonchus contortus* intestine: a prominent source of mucosal antigens. Parasite Immunol..

[60] Wang C, Li F, Zhang Z, Yang X, Ahmad AA, Li X (2017). Recent research progress in China on *Haemonchus contortus*. Front Microbiol..

