# Supplementary material for: A daf-7-related TGF-β ligand (Hc-tgh-2) shows important regulations on the development of Haemonchus contortus
Source: Parasit Vectors. 2020 Jun 26;13:326. doi: 10.1186/s13071-020-04196-x (PMC7318536; doi:10.1186/s13071-020-04196-x)
Supplement: Supplementary file 1 — Additional file 1: Table S1. Oligonucleotide primers (5’-3’) used in the present study. [file 13071_2020_4196_MOESM1_ESM.docx]

**Additional file 1: Table S1. Oligonucleotide primers (5’-3’) used in the present study.**

| **Primer** | **Sequence (5’-3’) ^a^** |
| --- | --- |
| Primers for real-time PCR | |
| Hc-tgh-2-rtF | GACGATTAGGCGACTCACG |
| Hc-tgh-2-rtR | TGGAATTCAACAAACGCATCT |
| Hc-tub8-9-rtF | TGTTCCATCACCCAAGGTATCC |
| Hc-tub8-9-rtR | TGACAGACACAAGGTGGTTGAGAT |
| Hc-18s-rtF | AATGGTTAAGAGGGACAATTCG |
| Hc-18s-rtR | CTTGGCAAATGCTTTCGC |
| Primers for RNA interference | |
| Hc-tgh-2-sF1 | **TAATACGACTCACTATAGGG**CTTCCGAGATCTACAATCGA |
| Hc-tgh-2-sR1 | GGATCCTCAGGAGCACGTACATTTAC |
| Hc-tgh-2-sF2 | GGATCCCTTCCGAGATCTACAATCGA |
| Hc-tgh-2-sR2 | **TAATACGACTCACTATAGGG**TCAGGAGCACGTACATTTAC |
| Bt-cry1Ac-sF1 | **TAATACGACTCACTATAGGG**CCAATACAGTACCAGCTACAG |
| Bt-cry1Ac-sR1 | GGATCCGATTCGGCTCTCCACAC |
| Bt-cry1Ac-sF2 | GGATCCCCAATACAGTACCAGCTACAG |
| Bt-cry1Ac-sR2 | **TAATACGACTCACTATAGGG**GATTCGGCTCTCCACAC |
| Primers for prokaryotic expression | |
| Hc-tgh-2-eF | CGCGGATCCGGTATGCAGGAGCCACCAAA |
| Hc-tgh-2-eR | CCGCTCGAGCTTGTCTTCTGGCATGCAGAC |

^a^ Underscore represents restriction sites and boldface represents a T7 promoter site.

Hc, *Haemonchus contortus*; Bt, *Bacillus thuringiensis*.
