# Supplementary figures and images for: A daf-7-related TGF-β ligand (Hc-tgh-2) shows important regulations on the development of Haemonchus contortus
Source: Parasit Vectors. 2020 Jun 26;13:326. doi: 10.1186/s13071-020-04196-x (PMC7318536; doi:10.1186/s13071-020-04196-x)

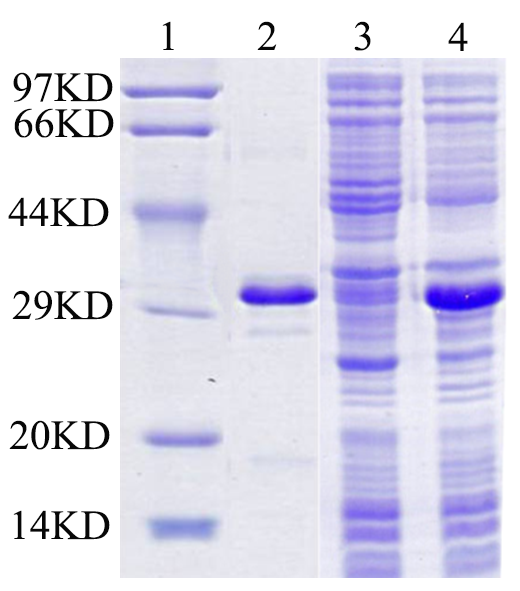

Supplement: Supplementary file 2 — Additional file 2: Figure S1. Prokaryotic expression and purification of Hc-TGH-2, as analyzed by SDS-PAGE. Lane 1: protein marker; Lane 2: purified recombinant protein Hc-TGH-2; Lane 3: recombinant protein expression non-induced by IPTG; Lane 4: recombinant protein Hc-TGH-2 expression induced by IPTG. [file 13071_2020_4196_MOESM2_ESM.tif]

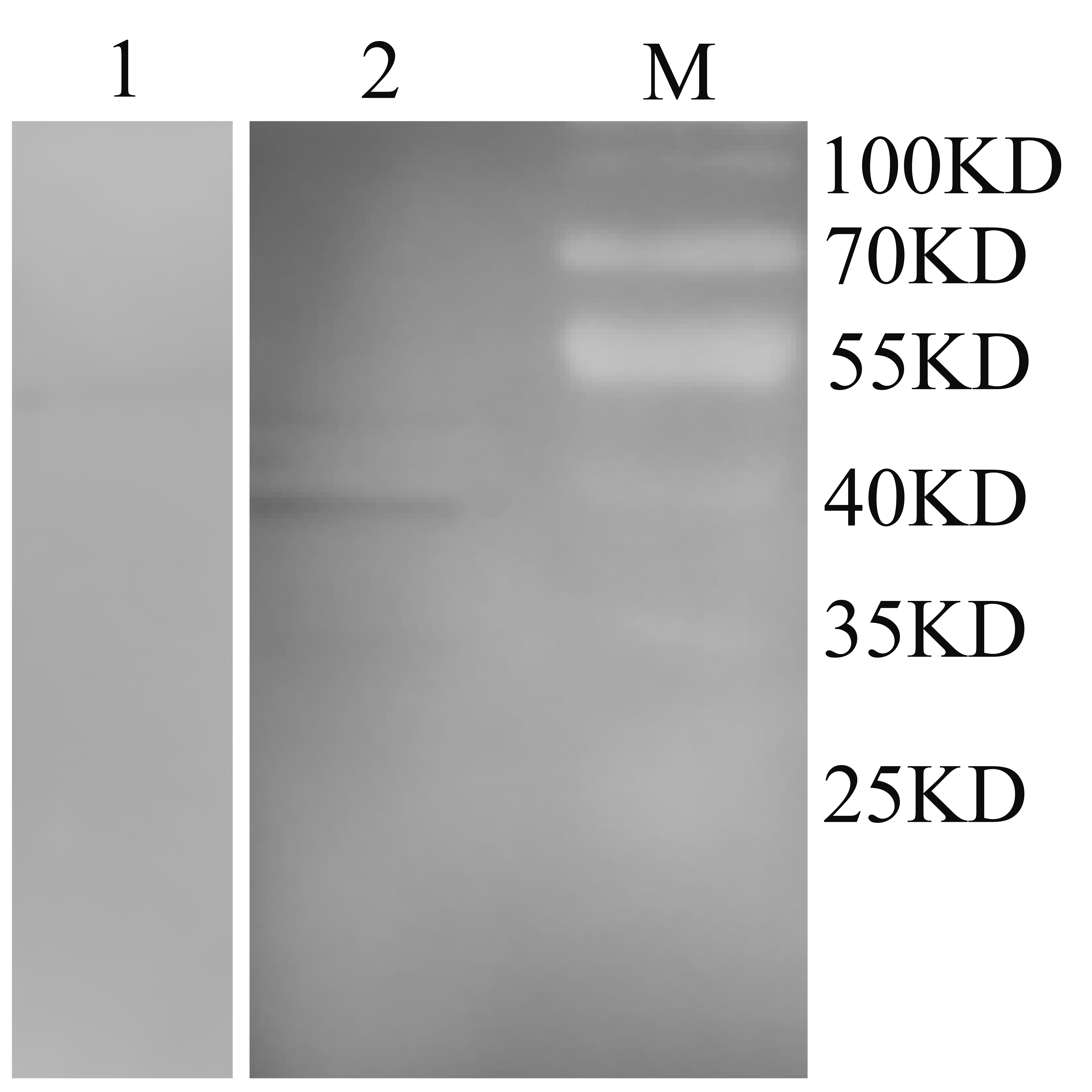

Supplement: Supplementary file 3 — Additional file 3: Figure S2. Western blot analysis detecting the expression of native Hc-TGH-2 in Haemonchus contortus adult worms. The polyclonal antibody of recombinant Hc-TGH-2 protein were analyzed by western blot using the total protein of Haemonchus contortus adults. Lane 1: negative serum without the antibody against recombinant Hc-TGH-2; Lane 2: positive serum with the antibody against recombinant Hc-TGH-2. [file 13071_2020_4196_MOESM3_ESM.jpg]
